# Reactive Exercises with Interactive Objects: Interim Analysis of a Randomized Trial on Task-Driven NMES Grasp Rehabilitation for Subacute and Early Chronic Stroke Patients

**DOI:** 10.3390/s21206739

**Published:** 2021-10-11

**Authors:** Andrea Crema, Ivan Furfaro, Flavio Raschellà, Mauro Rossini, Johannes Zajc, Constantin Wiesener, Walter Baccinelli, Davide Proserpio, Andreas Augsten, Nancy Immick, Sebastian Becker, Matthias Weber, Thomas Schauer, Karsten Krakow, Giulio Gasperini, Franco Molteni, Michael Friedrich Russold, Maria Bulgheroni, Silvestro Micera

**Affiliations:** 1 Translational Neural Engineering Laboratory, Center for Neuroprosthetics, Campus Biotech, EPFL, 1202 Geneva, Switzerland; ivan.furfaro@epfl.ch (I.F.); flavio.raschella@gmail.com (F.R.); 2 Centro di Riabilitazione “Villa Beretta”, Unità Complessa di Medicina Riabilitativa Ospedale Valduce, 23845 Costa Masnaga, Italy; ing.maurorossini@gmail.com (M.R.); fkt.davide.proserpio@gmail.com (D.P.); ggasperini@valduce.it (G.G.); fmolteni@valduce.it (F.M.); 3 Ottobock HealthCare Products GmbH, 1110 Vienna, Austria; Johannes.Zajc@ottobock.com (J.Z.); michael.russold@ottobock.com (M.F.R.); 4 Technische Universität Berlin, Fachgebiet Regelungssysteme, 10587 Berlin, Germany; wiesener@control.TU-berlin.de (C.W.); schauer@control.tu-berlin.de (T.S.); 5 ABACUS s.r.l., 20155 Milan, Italy; walterbaccinelli@ab-acus.eu (W.B.); mariabulgheroni@ab-acus.com (M.B.); 6 Asklepios Neurological Clinic Falkenstein, 61462 Königstein-Falkenstein, Germany; a.augsten@asklepios.com (A.A.); n.immick@asklepios.com (N.I.); k.krakow@asklepios.com (K.K.); 7 HASOMED GmbH, 39114 Magdeburg, Germany; sebastian.becker@hasomed.de (S.B.); matthias.weber@hasomed.de (M.W.); 8 The BioRobotics Institute and Department of Excellence in Robotics and AI, Scuola Superiore Sant’Anna, 56025 Pontedera, Italy

**Keywords:** sub-acute, stroke, interactive, grasp, rehabilitation

## Abstract

Enriched environments and tools are believed to promote grasp rehabilitation after stroke. We designed S2, an interactive grasp rehabilitation system consisting of smart objects, custom orthoses for selective grasp constraining, and an electrode array system for forearm NMES. Motor improvements and perceived usability of a new enriched upper limb training system for sub-acute stroke patients was assessed in this interim analysis. Inclusion criteria: sub-acute stroke patients with MMSE>20, ipsilesional MI>80%, and contralesional MI<80%. Effects of 30-min therapy supplements, conventional vs. S2 prototype, are compared through a parallel two-arms dose-matched open-label trial, lasting 27 sessions. Clinical centres: Asklepios Neurologische Klinik Falkenstein, Königstein im Taunus, Germany, and Clinica Villa Beretta, Costa Masnaga, Italy. Assessment scales: ARAT, System Usability, and Technology Acceptance. Methodology: 26 participants were block randomized, allocated to the study (control N=12, experimental N=14) and underwent the training protocol. Among them, 11 participants with ARAT score at inclusion below 35, n = 6 in the experimental group, and n = 5 in the control group were analysed. Results: participants in the enriched treatment group displayed a larger improvement in the ARAT scale (+14.9 pts, $p_{val}=0.0494$). Perceived usability differed between clinics. No adverse effect was observed in relation to the treatments. Trial status: closed. Conclusions: The S2 system, developed according to shared clinical directives, was tested in a clinical proof of concept. Variations of ARAT scores confirm the feasibility of clinical investigation for hand rehabilitation after stroke.

## 1. Introduction

Stroke is the leading cause of disability in developed countries. Impairment affects the large majority of stroke survivors and most of them require rehabilitation. Effective treatment is timely required to avoid the learned non-use of the affected arm [1,2]. Increased life span in developed countries, and lowered averaged age of first stroke translate into higher occurrence of stroke, longer disability-adjusted life expectancy, and higher cumulative post-stroke assistance [3,4]. More recently the COVID-19 pandemic caused a surge in the stroke population [5,6,7] and is expected to substantially modify the scenarios of treatment and social care [8]. If not treated properly, stroke survivors need constant external assistance even in basic daily activities. Environmental and personal factors can hugely affect patients’ reactions and expectations [9,10] in the acute and sub-acute phase.

Upper-limb paresis is the most common of a sequelae of impairments following a stroke, yet an effective treatment able to counter sensation of pain, feeling of foreignness, muscle weakness, and spasticity is still an open issue. NeuroMuscular Electrical Stimulation (NMES) is one of the treatments used to revert the learned non-use of the affected limb, and to improve grasp capabilities [3] in severe hemiplegic patients. However, current NMES systems have shown important limitations during unsupervised clinical use [11]. In a previous work [12] we designed a custom wearable device able to induce different types of hand grasps by selectively stimulating extrinsic and intrinsic hand muscles. This was possible due to the exploitation of spatial resolution features of the electrode array on extrinsic and intrinsic hand muscles.

We designed an improved version of our device to comply with updated clinical and technological requirements, and to support unsupervised task-driven clinical tests. A preliminary test with a cohort of sub-acute stroke survivors was performed in two clinical centres.

Historically, NMES systems have been tested primarily with chronic patients whereas studies on patients in the acute and sub-acute phases are more limited [13,14]. The proximal to distal gradient of motor and sensory deficits, and its longitudinal evolution after the event, do also affect the statistical availability of treatable patients in the conventional time windows for sub-acute and chronic stroke. For these reasons, the correct allocation of treatment of the distal segments is not necessarily obvious within the personalized clinical picture of the patient. However, post-stroke brain’s enhanced sensitivity to external treatments suggests that the anticipation of a treatment may result in better improvement for the patients. In particular, it is hypothesized that early treatment may provide improved motor rehabilitation over conventional chronic treatment because it avoids the learned non-use phase, and because treatment is provided in a recovery phase dominated by high cortical plasticity. However, enriched grasp exercises designed for chronic patients’ treatments are usually not suitable for the sub-acute phase.

In the next sections we describe the design process, the technological outcome, and clinical outcome of the interim analysis. Specifically, in Materials and Methods, we first describe the methodology for the iterative design and validation with clinicians, caregivers, and patients. The interaction of the central controller with orthoses, electrodes, stimulator, and environmental sensing elements is described, alongside with the modality of operations of each subcomponent. The description of a training session is then introduced. Finally, we analyse the interim data both in terms of patients’ outcome, and through a usability analysis of the whole assembly. One of the clinics involved in previous trials [12,15] is taken as a benchmark for expert clinics with solid technical and technological know-how; a second clinic, not previously exposed to similar technologies is used as a benchmark for clinics with limited confidence with these prototypes. In the results, we show global and treatment-specific motor improvements in patients. Moreover, we extrapolate indicators suggesting that the challenge posed by an exercise is a determinant factor in the perceived usefulness of the described device.

## 2. Materials and Methods

### 2.1. Methodology for the Design of the System Concept

The neuroprosthetic system was designed to rehabilitate hand functions through passive and active components. Passive wearables allow selective wrist and finger motion, and Electrode Arrays (EAs) induce controlled hand opening and closing via NMES. User-centred methodologies were used throughout the design phase; usability requirements were updated and refined during each development and verification phase. Physicians and neurologists, directly involved in the project, provided high-level clinical specifications; the bioengineering unit of one clinic further refined technical specifications. Two clinical focus groups—one with physicians not directly involved in the project, and one with patients and caregivers—were provided with an overview of the proposed system via a presentation and demonstration of looks-like prototypes of the sensorized orthoses. Questions raised during the demonstration were further addressed by the research technical staff and the hospital technical staff provided the updated requirement lists. In further steps, therapists suggested further changes. The resulting sub-components—Wearable NMES Orthosis, Graphical User Interface (GUI) for Virtual Electrodes (VE), Control Interface (CI), smart objects for environmental interactivity, Embedded Control System (ECS), and stimulation apparatus—are detailed in the following sections.

### 2.2. Technological Implementation

**Figure 1:**
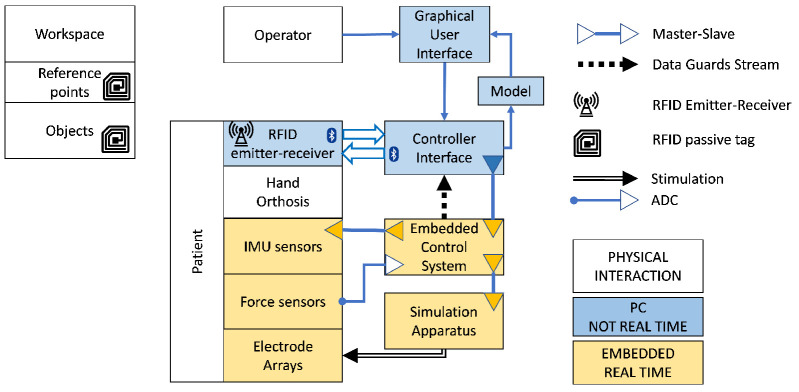
Architecture of the system. The prototype operates in three different domains (i) environmental interactivity (white), (ii) non-real-time control (teal), and (iii) real-time control (yellow). Devices in the real-time domain operate synchronously at fixed frequency with no jitter. Non real-time components operate at different frequencies aimed at giving a sensation of responsiveness but absence of jitter is not guaranteed. The controller interface (CI) operates as an exercise controller, logger, GUI for operators, and information broker from objects (Bluetooth) and RealTimeData. A multilayer master–slave architecture combines non-real-time components (CI) with real-time components managed by the Embedded Control System (ECS). ECS and CI share data and commands with a custom instruction set over UDP. The ECS controls the set of IMUs via I2C, and the stimulation apparatus via Virtual COM Port, and an on-board ADC for sampling grasp forces. Environmental interactivity is dependent on synchronous real-time information and control fed at 40 Hz (hand kinematics, thumb contact force, and pulse by pulse stimulation), and non real-time information (3–5 Hz) from RF passive tags.

The wearable NMES system uses topographically mapped multiple EAs, and acts as a modular tool usable for grasp rehabilitation. Three independent EAs are positioned on the forearm target extrinsic grasp muscles. An external stimulator with demultiplexers delivers stimulation patterns that can be manually tuned to elicit functional grasp, to obtain whole muscle conditioning, and to produce open-loop or closed-loop grasp control. Custom orthoses selectively constrain fingers and wrist, grasp force and hand kinematics are estimated through force and inertial sensors. The system interacts with daily-life objects able to supply information (e.g., the object’s physical characteristics, expected sequence of use) to drive their usage. The objects are equipped with radiofrequency sensitive passive tags, and a reader embedded in the system processes the associated information in order to run predefined sequences of actions. A devoted processing of the signal strength received from the tags and environmental constraints allows the recognition of the selected objects among others. The device control software is implemented in two main elements: (i) a master CI operates on a windows tablet, and (ii) a real-time compliant ECS implementing a deterministic state machine aimed at the control of the stimulation apparatus and of the wearable NMES hand orthosis. This concept is exploited to drive rehabilitation exercises to obtain deeper knowledge on the recovery of the patient. Figure 1 depicts an overview of the architecture.

#### 2.2.1. Wearable for NMES and Object Interactivity

**Figure 2:**
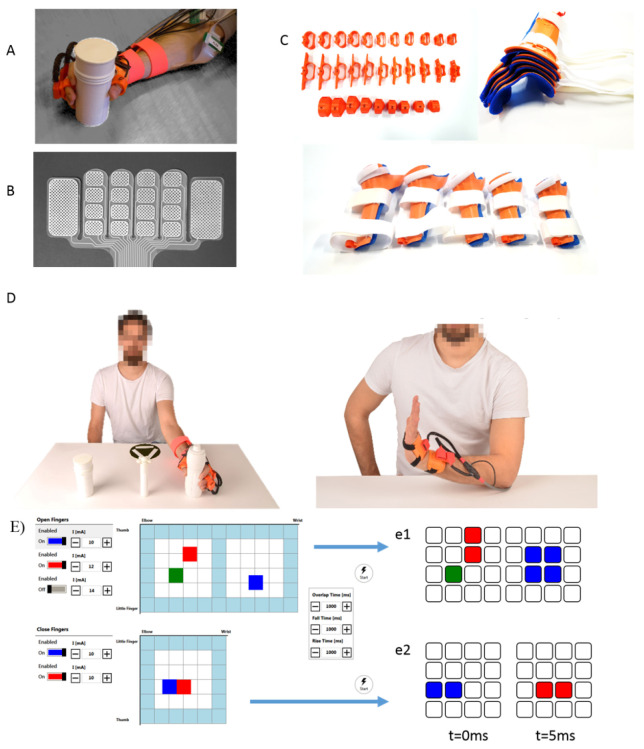
Wearable NMES system. The wearable system consists of units aimed at providing NMES, at constraining hand and wrist, and at sensing environmental interaction. Panel (**A**) The orthosis constrains the dorsal aspect of hand and wrist, and hosts the RF antenna between thumb and index to improve reliability and repeatability of object detection. A soft ring constrains the thumb in opposition and includes a force sensor for assessing localised contact force. Panel (**B**) Electrode arrays used for the trials. Panel (**C**) Anthropometric variability is accounted for by design. The rigid wrist-constraining orthoses are designed in five sizes and, if warmed, can be adapted to patient-specific ergonomic needs. Rings, proximal clasp, and distal clasps are soft and produced with a larger variability. Panel (**D**) Visual and auditory cues in the GUI precede the desired exercise execution. Panel (**E**) Stimulation maps are defined by virtual electrodes location and intensity of stimulation. Virtual Electrodes(VEs) can be enabled alone or combined, to fine tune the desired motor response. e1: frame-wise distribution of the VEs as configured in the GUI for extrinsic extensors; e2: in-frame patterning for the displayed configuration of extrinsic flexors.

We updated a previous prototype [12] by included symmetric, reconfigurable components that can be dynamically mapped, and driven by a novel stimulator. The new system, visible in Figure 2, includes EAs that target hand extrinsic muscles, with active sites and reference electrodes symmetrically positioned for usability with left and right arm. The minimal electrode size was chosen to selectively target patients with small anthropometry. Independent electrodes acting as active sites are sized 10×12${\mathrm{mm}}^{2}$, and grouped in 4 by 4 arrays. Electrodes sized 20×40${\mathrm{mm}}^{2}$—acting as reference—are positioned on opposite sides. EAs can be composed to create larger structures of custom shape. Medical-grade silver is screen-printed on functionalised polyester substrates. Electrodes are shaped in four-by-one independent rows to improve local flexibility. High-tack gel, die cut to shape, provides mechanical and electrical contact between skin and electrode.

#### 2.2.2. Orthoses and Sensorized Components

Post-stroke flaccidity, appearance of spasticity, and onsets of pathological synergies [16] affect the execution of complex movement combinations. In sub-acute patients, variations across these stages can be frequent, and need to be accounted for on a per session basis. We designed modular constraining orthoses to accommodate the expected lack of hand control while preserving the grasp capabilities of the subjects, and the tactile afferences that patients rely on during grasp. The Type-A orthosis, visible in Figure 2 panel C, is aimed at constraining wrist and fingers control. Five sizes account for forearms ranging from 200 mm to 350 mm. The dorsal side of the forearm is covered for two thirds of its length, as well as the hand dorsum. In standard conditions the orthosis locks the wrist in extended positions at 15°, and sets the thumb in slight opposition to the other fingers. However, all rigid parts can be be heated with hot water and reshaped as needed. Rigid parts are foam padded (OttoBock, Plastazote) for improved comfort. Finger clasps and rings, made of thermoplastic rubbers, bind the fingers together and lock the thumb to the orthosis. Clasps and rings host Inertial Measurement Units (IMU) (TDK InvenSense, MPU9250), and force sensors (Tekscan Flexiforce, A201-A401). The Type-B orthosis, Figure 2 panel D, is only aimed at monitoring hand-wrist kinematics.

#### 2.2.3. NMES Controller

A GUI, hosted by the CI, represents sets of VEs associated with task-specific stimulation maps. Single VEs and complete stimulation maps can be personalised and tested in calibration mode. Location and intensity of stimulation to each VE is set on a touch responsive grid (Figure 2) mimicking the topological disposition of the electrodes on the skin. EAs target proximal hand extrinsic flexors, and proximal and distal hand extrinsic extensors. The NMES controller, hosted in the ECS, provides assistance as needed with dynamically adaptable spatial and temporal stimulation features. The location of the VE within the grid determines the activation profile of the corresponding physical electrodes by activating elements of size 1×1, 1×2, 2×1, or 2×2. The operator can personalise the motor response by continuously varying spatial and depth selectivity in the longitudinal and transversal axes. The maximum allowed current intensity of each VE can be adapted in increments of 1 mA while maximal pulse width and stimulation frame frequency are set to 300μs and 40 Hz. Within each frame, pulses and associated activation profiles are sequential to ensure independence of VEs, and to provide variable spatial and temporal patterning. This approach allows to target spatially-distinct or depth-distinct areas with appropriately distributed VEs, thus allowing to configure mixed-use cases. Kinematic and force signals are continuously measured; within the range of stimulation acknowledged as comfortable by the patient and in accordance to the motor recruitment needs, the pulse width was modulated between 50μs and 300μs. Each exercise subtask, when triggered by the GUI, enables personalized force or a position setpoints, and switches to the corresponding tuned Proportional Integral (PI) controller to modulate the stimulation to match the desired grasp force or kinematic profile. During transitions, the control is modulated for a soft-transition. The reason for this approach is twofold: (i) the slowly ramping setpoint prevents sharp stimulation increments; (ii) subjects are able to perceive the stimulation sensation before the motor recruitment onset, and if capable of performing the specific subtask, can complete the task autonomously. This approach is aimed at limiting subjects’ slacking behavior, which reduces the patient involvement in the task, and ultimately inhibits motor re-learning [17]. Patients unable to perform the desired movement volitionally do still benefit from this approach as stimulation is modulated to reach the desired fingers’ extension or grasp.

#### 2.2.4. Stimulation Apparatus

The stimulator RehaMove Pro [18] is a miniaturised, configurable, prototypical system for neuromuscular electrical stimulation via surface electrodes. The stimulation can be using an application programming interface (API) and library called ScienceMode, which allows defining stimulation currents up to 130mA, pulse widths of 20–16,000 μs and frequencies of 1–500 Hz. Stimulation voltage is limited at 150V. The stimulator weighs 280g and measures 50mm×73mm×32mm. A connectable unit enables us to apply stimulation currents via a multi-electrode array by de-multiplexing a stimulation channel. The demux used in this prototype can support up to 48 independent active sites and 3 counter-electrodes. After testing, the stimulator voltage has been limited to 90V.

The stimulator RehaMove Pro [18] is a miniaturised, configurable, prototypical system for neuromuscular electrical stimulation via surface electrodes weighting 280g and measuring 50mm×73mm×32mm. The stimulation is controlled through a Virtual COM port through a custom communication library. A connectable unit enables the application of stimulation currents via a multi-electrode array by de-multiplexing a stimulation channel. The demux used in this prototype can support up to 48 independent active sites and 3 counter-electrodes, with stimulation voltages up to 90V

#### 2.2.5. RFID System for Environmental Interactivity

The RFID module acknowledges the CI that a certain object or a position in the space has been reached by the patient. The system is composed by three elements: a radio-frequency emitter-receiver (qIDmini R1170I, CAENRFID) with an external antenna positioned on the hand orthosis, objects/positions equipped with three passive tags each, and a back-end to manage the operations and elaborate the signal. For each tag, identified through its Unique IDentifier (UID), the Received Signal Strength Indicator (RSSI) measures the power of the backscattered signal. Functional elements in the physical workspace were labelled with programmable RFID tags, so that the signal received from a tag UID is associated with a specific smart object. The RSSI of each target tag is used as an estimator of the relative proximity between a smart object and the patient’s hand on which we mounted the antenna. Tags available in the operational range of the reader are retrieved and contextually filtered. For each instant, the RSSI value of the matching tags is extrapolated and weighted to provide spatial filtering. For each detectable object the five most recent frames of RFID information, approximately equal to one second, is averaged. The target is labelled as reached when the signal strength exceeds the corresponding threshold.

#### 2.2.6. Control Interface and Embedded Control System

The CI controls the modules not requiring real time constraints. It comprises a GUI, a database and a finite state machine to perform training sessions. The GUI is designed as a step-by-step wizard, guiding therapist and patient through the steps required by the training session: configuration (adapting the system to the patient’s needs), donning, parametrization (stimulation and state machine settings), training execution. The GUI is designed to be touch-compliant and to operate on a tablet PC (Surface 3 Pro, Microsoft Corporation, Redmond, WA, USA). The ECS controls all the modules requiring real time constraints, such as the stimulator, the NMES controller and the IMU and force sensors. To keep the CI and the ECS synchronised, a strict master–slave concept using a custom-made communication protocol based on User Datagram Protocol (UDP) was implemented. The ECS is powered with one single medical power supply and all sensors of the orthosis are connected via one DSUB 25 plug. Inside the ECS a Beaglebone Black runs a standard Debian Linux kernel. A customised embedded real-time target for the Simulink Coder (Mathworks, Natick, MA, USA) is used to generate the real-time code for the experiment. Transitions between states are triggered upon reception of new sensor data (RFID, IMU, FSR), but only executed if predefined conditions are met.

#### 2.2.7. Description of a Training Session

Each patient of the experimental group was assigned with a personal kit of electrode arrays, and of orthoses of the appropriate size, which could be further shaped as needed. A typical training session consists of four main phases: the setting, donning and parametrization of the system, and the training which consists of a sequence of selectable exercises. The GUI supports the therapist and the patient through all the phases.

In the setting phase, the therapists created a new user or selected an existing one, and selected the desired exercise and its duration, while in the donning phase they positioned the electrode arrays on the patient’s forearm.

During the donning phase of the first session, rigid orthoses of appropriate size were heated and shaped to match the patient characteristics. If the exercise were aimed at the wrist motion consolidation, the Type-B orthosis was donned. For the exercises requiring fingers motion consolidation, or for grasp exercises with the objects, the Type-A orthosis was required. In this case, after checking the electrode positioning, the rigid forearm–hand part was first stabilised with Velcro on the hand palm, then on the wrist, and finally at the proximal end.

In the calibration phase, stimulation maps set through a GUI walkthrough. Each map, containing the stimulation location and intensity for the VEs, was updated as needed at the beginning of each session. Similarly, the desired kinematic thresholds in flexion and extension for the fingers, or for the wrist, and for the desired grasp force for each object were set. The touch interface allowed dragging of the VEs and the immediate application of the new configuration, therefore the therapist not only verified the efficacy of the stimulation, but also its perceptual acceptability. This approach also allowed the patient to self-calibrate the stimulation in an easy and understandable fashion. The most recent calibration parameters for the sensors and for the stimulation maps were stored on the GUI as templates. This approach allowed us to speed up the setup in the following sessions if the motor response did not differ significantly.

Training consisted of exercises involving the reaching, hand opening, grasping, moving objects (small, medium, or large) in a plane, and releasing of the objects. Exercises could include the above-mentioned tasks, or a subset. Moreover, exercises were stratified to provide incremental complexity. A GUI controlled the exercises’ execution, and allowed the therapists to supervise the single tasks both visually and with audio.

### 2.3. Clinical Test on Sub-Acute Stroke Patient

This interim clinical test is part of a larger multicentre RCT of the RETRAINER project, in which a lightweight arm exoskeleton (RETRAINER S1) and the hand orthosis (RETRAINER S2) were tested. The S1 and S2 subsystems are considered for a future integration, and thus tested on populations sharing the same inclusion criteria. This set of preliminary tests of the open-label parallel two-arms trial specifically refers to the RETRAINER S2 prototype, tested at Asklepios Neurologische Klinik Falkenstein, Königstein im Taunus, Germany, and at Clinica Villa Beretta, Centro Complesso di Neuroriabilitazione, Ospedale Valduce, Costa Masnaga, Italy. Participants were recruited directly by the clinicians from the inpatient population. In Italy the study was approved by “Comitato etico interaziendale delle provincie di Lecco, Como, e Sondrio”, protocol number 0019737/16U, and by the Italian Ministry of Health (protocol number 0022261 27/04/2016 DGDMF COD UO-P). In Germany the study was approved by Ethik-Kommission bei der Landesärztekammer Hessen, tracking number III/1/sja/bog FF 29/2016. The participants provided written informed consent before participation and consented to the publishing of their collected data.

#### 2.3.1. Inclusion Criteria

**Figure 3:**
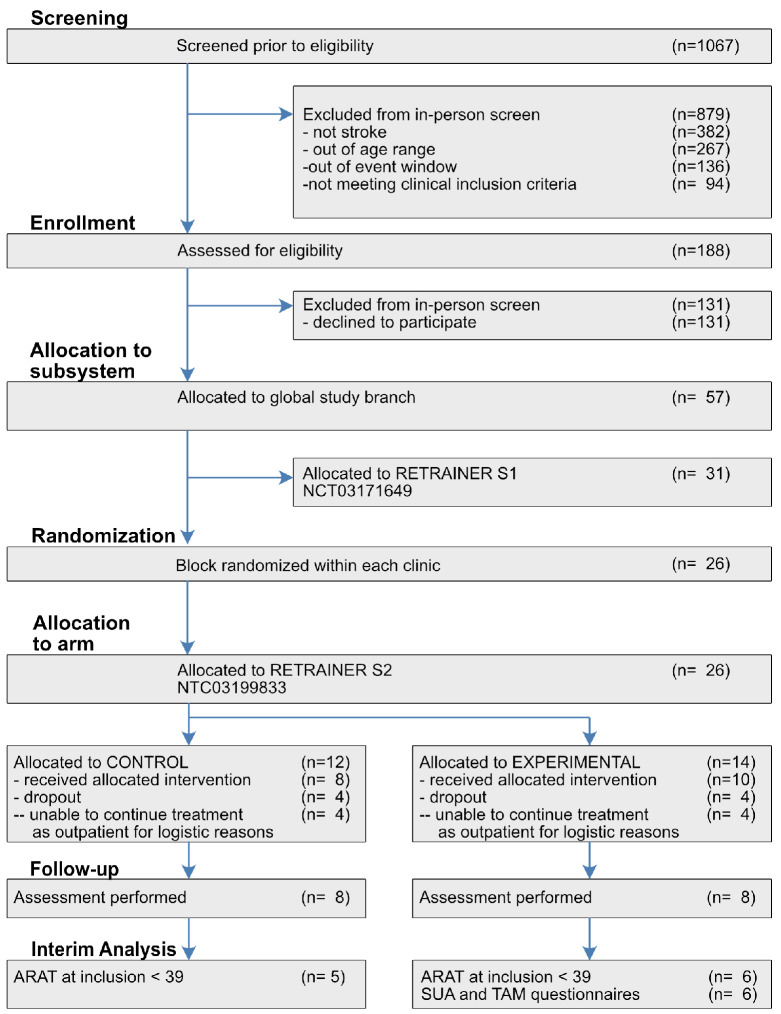
CONSORT diagram. Consolidated Standards of Reporting Trials diagram. Depiction of subject selection, group allocation, attrition, and data analysis. Abbreviations: S1, subsystem 1; S2, subsystem 2; CONTROL, S2 control group; EXPERIMENTAL, S2 experimental group.

Patients who have suffered a first stroke with major unilateral functional impairment, aged between 18 and 85 years, male or female, can be enrolled in the study between two weeks and nine months after the acute event. The Motricity Index [19] of the affected side must be under 80% of best expected performance with no major contra-lateral impairment. Residual muscular activity for forearm muscles (MRC>0) [20], and sufficient cognitive contextual capabilities (MMSE>20) [21]. Limitations for using the device due to impairment of Passive Range of Motion and/or pain due to Spasticity are exclusion criteria, as well as previous history of major neurological or psychiatric disorders or allergy to electrodes. In each hospital, after preliminary screening, Figure 3, patients are assigned to S1 or S2 subsystems on the basis of their clinical conditions and rehabilitation target. A parallel dose-matched two-arms trial, conventional versus experimental treatment, is used to differentiate the effects of the subsystem under test. The expected number of patients (n = 68) for the S2 trial is based on the empirical expectation of the target population available in the two clinical sites in a time frame of two years. The numbers of participants assigned to each group is kept similar over time through a randomized sequence. The assignment to the conventional or S2 experimental branch follows a block randomization compliant to Kang [22] (block size = 4). Among all the possible balanced combinations of assignments within the block (n = 6), a dice roll is used to select the local block sequence. Before the trial started, an engineer of the Italian clinical site generated a randomized sequence for each clinical centre. Clinicians of both sites, blind to the randomized sequences, enrolled participants. Finally, the engineer assigned each new participant to the corresponding intervention. In this paper, we performed an interim analysis on the results associated with the first time frame of the study, aiming to include at least 5 participants per group.

#### 2.3.2. Description of Exercises

There is a relative availability of tools for motor rehabilitation after stroke, but most of them are not aimed for NMES-assisted contextual interaction. Exercise design is in conformity to community guidelines [23] suggesting task-oriented training approach consistent with the WHO-ICF [24] in terms of function level, activity and participation level. In this perspective, the system is designed to provide exercises that can be performed in a natural environment, with task-specific training, tailored on the needs of the subject, and meaningful for the specific functional goals. Two functional areas are targeted: potentiation of wrist and fingers flex-extension, and progressive functional object-grasping. Tasks of increasing complexity are aimed at providing sufficient exercise variety for patient, and yet to avoid decision-making paradox for therapists. In the first set of exercises the patient’s arm is supported by a cushion or a wheelchair arm support, no spatial exploration takes place, and the patient’s attention needs to be focused only on hand opening and hand closing. In this case NMES is controlled only by IMUs to produce flexion and extension of wrist and fingers in accordance with the desired movement profile. As described in Section 2.2.3 the patient is informed about the desired movement by visual and auditory cues. The NMES controller provides a progressive stimulation profile which implicitly informs the patient of the desired movement onset and modulates the intensity to compensate for kinematic profile mismatches. With this approach, multimodal cues are associated with the execution of a simple task, and reinforcement learning is provided through the repetition of the task. Visual or auditory cues can be selectively suppressed by the operator, leaving NMES as the only explicit cue. In the second set of exercises patients use the same learning scheme, but are also required to actively interact with the environment. In this case, the functional compliance of the treated limb must be guaranteed by the motor control of the subject or with external support. Patients with localised flaccidity or spasticity that prevent wrist–hand support and thumb opposition need to use the rigid orthosis to lock the hand–wrist position, and optionally to fix the thumb in opposition.

Four different exercises are implemented: (i) flexion and extension of fingers; (ii) grasp and release cylindrical objects with different sizes and weights; (iii) grasp, move, and release objects on a plane; (iv) grasp, move at shoulder level, and release objects in space. In exercises (iii) and (iv) the subject is seated in front of a desk with the height to have the elbow at 90° of flexion and no compensation of shoulder in frontal plane; three positions are chosen within the workspace reachable by the patient; an object has to be reached, grasped, and moved to the chosen position and released each time; after each drag and drop sequence the subject returns in the rest position. Counter gravity lifting is required in the most complex scenario. The mix of exercises is chosen by the supervising clinician according to the residual functional ability of each subject and the rehabilitation goals, and can be adapted in order to follow the evolution of the subject.

It is particularly important to notice that central fatigue and perceived peripheral fatigue play a central role in the willingness to focus on targeted exercises execution, as well as specific dissociations between motor control, body representation, and sensorial afferences. The supplemental treatment with S2 may allow exploring combinations of treatments, or intensities of treatment not obtainable with the conventional mix of technologies. Hence the exercise selection may compensate for “feeling of early exhaustion” and “aversion to effort” [25] and allow for extended meaningful training.

#### 2.3.3. Treatment Structure

The experiment consists of twenty-seven treatments over nine weeks. Each treatment provided to the patients includes one hour of conventional treatment. The conventional treatment can include a combination of the following treatments: upper limb passive motion, arm cycle ergometer with or without NMES, NMES on non-target segments, upper limb exercises using augmented-reality or virtual-reality, occupational therapy, constraint induced movement therapy, upper limb active movement (reaching, grasping, elevation, spatial orientation), repetitive task training, mirror therapy, writing training, and chemodenervation therapy. S2 orthoses and clasps are not used for the conventional treatment. Participants assigned to the control group receive an additional treatment of 30 min per session, whereas participants assigned to the experimental group perform exercises with the above described prototype. A test was conducted to validate the functionality of the system, verify its usability in a clinical environment, and to have an interim assessment of the potential outcome of the treatment. Table 1 reports the demographic and clinical details of the participants. As characterised by the Action Research Arm Test (ARAT), participants at inclusion had different levels of impairment, with performances of the affected side similarly distributed among the control and the experimental group.

#### 2.3.4. Clinical Outcome Assessment

The functionality of the whole arm (ipsilesional MI≥80%, contralesional MI≤80%) was chosen to harmonise the inclusion criteria of S2 to S1. For reference, please check Figure 3. However, this criterion—while fit for S1—did not appear sufficient to separate patients with almost intact grasp capabilities from more impaired ones, as the MI targeted mostly reaching functionality which was the aim of S1. Here, to more appropriately proceed with assessment, we restricted the analysis to patients with ARAT score at inclusion lower than 39; this threshold was chosen to eliminate the patient mostly affected by score ceiling. As a preliminary descriptor of the training effects, the consortium decided to verify differences between the groups with at least 5 patients per group.

We tested whether the different treatments were effective in ameliorating the motor functions, and if any improvement varied as a function of the initial conditions and of the treatment. Patients’ scores at the primary motor outcome (ARAT Total, Pinch, Grip, Grasp, and Gross Arm Movements) were collected during the assessments at inclusion (week 0, T0), end of treatment (week 9, T1), and follow up (week 13, T2). ARAT score and sub-features were compared between the two groups of patients with a linear model to predict score outcome using initial conditions and treatments as fixed factors and patients as random factors. Model acceptance, $r^{2}$, and p-values were obtained through F-test versus the null model; likewise, coefficients for global score changes, and group-specific changes were fitted through ordinary least squares, and p-values obtained from t-statistic. The model, fitted on the longitudinal assessment data according to the formula $Tb∼c_{Ta}*Ta+c_{C}+c_{R}$, estimated the global variation ratio from $T_{a}$ to $T_{b}$ with coefficient $c_{Ta}$, and group specific effects with $c_{C}$ and $c_{R}$. The statistical analysis was performed with StatsModels version 0.12.2 [26].

#### 2.3.5. Usability Assessment

The system here described is a prototype system to evaluate the technical concept, which of course can not provide the usability of a commercial device. However, Likert questionnaires were used to benchmark the perceived usability of the system. Patients training with the prototype evaluated the whole system at the training completion (T1). The assessment of the prototype was performed in a two steps process. Participants were asked to complete the System Usability Scale questionnaire (SUS) [27,28]. For a more in-depth evaluation of the different factors affecting the perceived quality of the system and its acceptance, a Technology Acceptance Model (TAM) [29,30] was also used. Basic descriptive statistics, and cross-correlations (Pearson’s r) were calculated for these measures.

## 3. Results

### 3.1. ARAT Score

Between 11 April 2016 and 1 December 2018, 24 patients were enrolled; in each arm 4 patients were unable to continue the treatment as outpatients for logistic issues and 16 patients completed the training. The interim analysis presented here focuses on 11 patients who met the supplemental conditions of ARAT score at inclusion not subject to ceiling effects (C: control, N=5 E: experimental, N=6). Out of the 11 patients’ scores here reported, 2 belong to the more expert clinic. No adverse effect was observed in relation to the treatments.

The minimal clinically important difference (MCID) of the total ARAT score in chronic stroke patients is approximated to 6pt, roughly equal to 10% of the scale. According to Lang [31], in sub-acute stroke patients the patient-perceived MCID has to be considered of 12pt for the dominant side, or 17pt for the non-dominant side, representing 21% and 30% of the scale, respectively).

Between the two groups, differences in age, delay of intervention after stroke onset, and ARAT level at inclusion were not significant (*t*-test for independent samples, $p_{\mathrm{age}}=0.293$, $p_{\mathrm{delay}}=0.245$, $p_{\mathrm{ARAT}}=0.850$). Patients ratios for affected side and stroke type were also similar (Fisher exact test, p=1 in both cases). Baseline analysis results are reported in Table 2.

**Figure 4:**
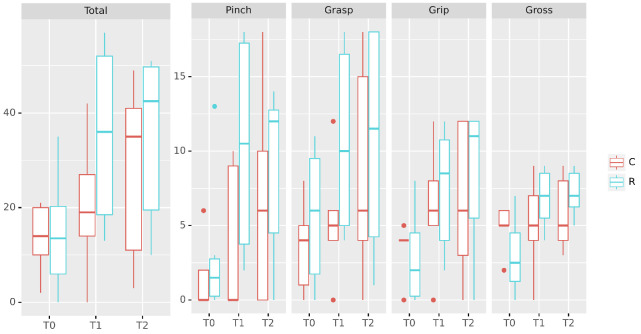
ARAT scores changes Results of treatment. Top: boxplot with patients scores for each ARAT subscale. Bottom: variations if each patient shown as a lineplot. Group assignment by hue; C: Control group, R:RETRAINER S2 group. Assessment performed at inclusion (T0), end of treatment (T1) at week 9, and follow-up assessment (T2) at week 13. The experimental treatment effect appears provide a significant onset within the in-treatment phase.

In general patients improved after the treatment for both groups. Assessment results for ARAT scale and sub-scales are summarised in Figure 4. Detailed mixed models and results are reported in Table 3. For ARAT total the global in-treatment effect (T1 vs. T0 model, $r^{2}$ = 0.746, F = 11.74, p=0.0042) resulted in an increment of baseline score of 39% (p=0.0028) and in an increment of 14.94 points (p=0.0494) specific to the experimental group. Considering the carry over effect (T2 vs. T1, $r^{2}$ = 0.856, F = 23.69, p=0.0004), 98% of the score at the end of treatment was retained (p=0.0002). The improvement was not specific to the pinch action ($r^{2}$ < 0.310, p>0.2260 for T1 vs. T0 and T2 vs. T0), showing that finer grasp capabilities improvements were volatile, and did not improve globally but a moderate improvement was specifically associated with the experimental group (+8.68 points, p=0.0262). Considering grasp in-treatment variations (T1 vs. T0 model, $r^{2}=0.738$, F = 11.28, p=0.0047), an improvement trend toward significance was estimated for the experimental group(+4.15 points, p=0.0898), but the main baseline improvement was global (+15%, p=0.0037). This improvement was persistent at follow-up (T2 vs. T1 model, $r^{2}$ = 0.882, F = 30.02, p=0.0002) showing a 29% global increase of the Grasp score (T2 vs. T1, *p* < 0.0001), but not associated to specific groups. Grip score displayed a trend to significance (T1 vs. T0, $r^{2}$ = 0.513, F = 4.222, *p* = 0.0560) for in-treatment proportional variations ( global increase +12%, p=0.0222) and specific improvements associated to the experimental group (+4.93 points, p=0.0371). During follow up, the grip model was significant (T2 vs. T1, $r^{2}$ = 0.758, F = 12.56, p=0.0034) and associated with a global improvement of 5%(p=0.0012), but not dependent on specific groups (all *p*-values > 0.8354). Gross movements score improved during the in-treatment phase (T1 vs. T0, $r^{2}$ = 0.545, F = 4.786, p=0.0431) increased in the experimental group (+4.29 points, p=0.0093) but not in the conventional group(p=0.6116), probably due to the volatility of some score in the control group.

### 3.2. SUS and TAM Scores

We proceeded assessing descriptive statistics and correlation of SUS and TAM scores at the end of treatment, and of the ARAT scores at inclusion. The SUS score, detailed in Table 4, shows a moderate perceived usability. The increase over time of the SUS score at the naïve clinic, not shown, suggests that the operators are learning to use the device, and thus the patient’s perception about the device are also affected by the confidence of the operator with the device. Technology acceptance scores did show that patients had mixed perceptions related to the full prototype. In particular, in this initial phase, characterised by learning the use of the device for both clinical operators and patients, the perceived usefulness was neutral (50% of the likert scale). Patients had limited perception (35.5%) of external control, finding the system usable for not tightly constrained exercises. Moreover, relatively high scores were reported for ease of use (69%) and enjoyment (78.5%). Strong correlations [32,33] with ∣ρ∣ > 0.8 and p<0.05 are reported. The ARAT score at inclusion had a strong negative correlation with the ease of use, meaning that patients with low score at inclusion found the device driven exercises easy to perform, and vice versa, patients with higher motor capabilities found it less usable. The enjoyment to use the system was positively correlated with the motor score at inclusion. SUS did not correlate with the other scales. Within-TAM correlations let emerge that patients who found the exercises easier to perform did consider them less useful, or that an exercise perceived as challenging is also perceived as more useful. The usefulness of the exercises was positively correlated with the perception of external control from the setup, and with the enjoyment of the activity. Moreover, the ease of use of the setup and of the exercises was negatively correlated with the enjoyment of the activity.

## 4. Discussion

Our neuroprosthetic system has been developed taking into account clinical suggestions and tested in a clinical proof of concept with two clinical centres. The results confirm the feasibility of using our system for the hand rehabilitation after stroke. All the participants were able to use the system, and through the SUS and TAM assessment judged positively the usability of the device. During the first training sessions, clinicians needed to understand how to effectively calibrate the sensors and to set up the system. SUS scores improved over time, suggesting that an improved confidence of the clinicians with the device perceived by the participants. The main concept of this experimental treatment is to help the subject in performing the grasping of the interactive objects of the setup, and to perform exercises mimicking ADLs. Therefore, the target group consists of stroke patients with limited grasp capabilities, and with a variable degree of wrist-hand support. The capability to control accurately the extended reaching of an object—that is to stabilize correctly the shoulder and control the elbow—is only required for a subset of the exercises, and the workspace is adaptable. Our system seems able to complement the conventional treatments. As the overall ARAT score improvement is higher in the experimental group than in the conventional treatment, at least for patients with moderate to severe grasp deficiencies, it would be worth assessing how a higher quantity of treatment, or the interaction with other enriched treatments would balance the apparent benefits.

The system here presented has some technological limitations. First, the electrode arrays could benefit from a more flexible material to have better localized adhesion with the skin, and longer tails acting as mechanical stress absorbers; the gel is also mechanically cut from the commercial roll, and the overall packaging design could be improved to ease the removal of the plastic layers used to prevent gel dehydration. Second, the stimulation of intrinsic hand muscles would allow training a variety of pinch grasps, now not possible. Third, ECS required ultra-low capacitance cables to communicate with the IMUs, requiring stiff cables. An ECS supporting reliably wireless sensors communication would avoid this problem, and improve overall lightness, usability, and reliability of the wearable. Additionally, the stimulator’s demux is not always able to sustain the required intensity of stimulation; sample cases are subjects with excessive adiposity—which acts as a high impedance—bad skin preparation, or badly connected wearables. Last, the engagement in the exercises is improvable with smoother environmental interactivity; since the RFID antenna should not obstruct the grasp, nor the user’s view, the current size of the RF antenna obliges to position it on the dorsal side of the hand; the hand thickness, in contrast, may shield the RF emission and response leading to a variable efficacy in object detection.

## 5. Conclusions

The framework of reactive exercises with interactive objects seem to offer adequately challenging training to patients with limited grasp capabilities. Results emerging from the interim analysis suggest that a larger recovery may be conditioned to the use of the described system as a supplement to the conventional treatment. One source of clinical disparity lies in the fact that each clinic used its own standard best set of treatments as conventional treatment, consequently potentially masking or enhancing the effect of this enriched treatment. The sample size was low and thus the reported results are not generalizable to the target population of sub-acute stroke survivors with upper-limb motor impairments. However, it is important to notice that a clinical study is ongoing, and the complete dataset of 68 patients is expected to provide more robust insights on the treatment effects. Future work to translate the research findings herein reported into clinical practice will require the deployment of the proposed system in the home setting and a rigorous evaluation of its robustness, usability, and clinical effectiveness. 

## Figures and Tables

**Table 1:** Demographic data of the participants in the control group (C) and in the experimental group (E). I: Ischemic. H: Hemorrhagic.

Group	Sex	Age (yr)	Days since Stroke	Affected Side	Stroke Type	ARAT T0
C	M	59	116	R	I	14
C	M	69	83	L	I	20
C	M	69	35	L	I	21
C	M	54	247	L	H	2
C	M	54	125	R	I	10
E	F	72	112	R	I	35
E	M	73	108	L	I	9
E	F	79	22	L	I	5
E	F	72	15	L	I	21
E	F	73	18	R	H	18
E	M	42	145	R	H	0

**Table 2:** Descriptive statistics of the interim analysis. SW: Shapiro Wilk. MW:MannWhitney. TT: *T*-test. F: Fisher exact test.

Scale	Label	Conv	Exp	Baseline Test	Stat	*p*-Value
Sample size		5	6			
Age		59 (54:69)	72.5 (72:73)	SW	0.8839	0.1168
				MW	5.0	0.0400
				TT	−1.1165	0.2931
Interv. delay		116 (83:125)	65 (19:111)	SW	0.8951	0.1607
				MW	8.0	0.1177
				TT	1.2433	0.2451
Affected side	L / R	3/2	4/3	F	0.75	1
ARAT Total	T0	14 (10:20)	13.5 (6:20.25)	SW	0.9513	0.6616
				MW	14.5	0.5
	T1	19 (14:27)	36 (18.5:52)			
	T2	35 (11:41)	42.5 (19.5:49.75)			

**Table 3:** ARAT score longitudinal analysis through mixed models. Model comparison reports the coefficient of determination, F score, and *p*-value. For each model coefficients are reported alongside with *p*-values. Significance: + 10%; * 5%, ** 1%, *** 0.1%.

Scale	Model Comparison	$r^{2}$	F	*p*	Group C Coeff	*p*	Group R Coeff	*p*	Global Recovery Coeff	*p*	Significance Model	C	R	Global
ARAT Total	T1∼T0	0.746	11.74	0.0042	1.77	0.7909	14.94	0.0494	1.39	0.0028	**		*	**
	T2∼T0	0.656	7.626	0.0140	8.71	0.2746	14.11	0.0943	1.42	0.0054	*		+	**
	T2∼T1	0.856	23.69	0.0004	7.77	0.1310	0.32	0.9592	0.98	0.0002	**			***
ARAT Pinch	T1∼T0	0.310	1.801	0.2261	2.97	0.3636	8.68	0.0262	0.52	0.3618			*	
	T2∼T0	0.133	0.6122	0.5660	5.92	0.0995	7.09	0.0637	0.55	0.3507		+	+	
	T2∼T1	0.601	6.027	0.0253	3.78	0.1317	0.62	0.8452	0.79	0.0094	*			**
ARAT Grasp	T1∼T0	0.738	11.28	0.0047	1.25	0.5188	4.15	0.0898	1.15	0.0037	**		+	**
	T2∼T0	0.472	3.572	0.0779	3.87	0.2730	3.23	0.4194	1.31	0.0309	+			*
	T2∼T1	0.882	30.02	0.0002	1.62	0.3363	−3.11	0.1863	1.29	<0.0001	***			***
ARAT Grip	T1∼T0	0.513	4.222	0.0560	2.38	0.2616	4.32	0.0371	1.12	0.0222	+		*	*
	T2∼T0	0.404	2.717	0.126	2.52	0.3713	4.93	0.067	1.20	0.0557			+	+
	T2∼T1	0.758	12.56	0.0034	0.06	0.9742	0.42	0.8354	1.05	0.0012	**			**
ARAT GrossM	T1∼T0	0.545	4.786	0.0431	0.93	0.6116	4.29	0.0093	0.85	0.0258	*		**	*
	T2∼T0	0.472	3.581	0.0775	2.92	0.0784	5.37	0.0008	0.60	0.0481	+	+	***	*
	T2∼T1	0.858	24.19	0.0004	2.26	0.0098	2.34	0.0222	0.71	0.0002	***	**	*	***

**Table 4:** Questionnaires analysis. Scores are reported as median and IQR; Spearman correlation between scales is reported for significant and close to significant results.

Measure	Median	IQR	Correlations SUS	TAM Usefulness	TAM Ease	TAM External Control	TAM Enjoyment	ARAT Total T0
SUS	55%	(52.5%:72.5%)	∖	//	//	//	//	//
TAM Usefulness	50%	(21.25%:67.5%)		∖	ρ = −0.8117	ρ = 0.8872	ρ = 0.9209	
					*p* = 0.0498	*p* = 0.0183	*p* = 0.0091	
TAM Ease	69%	(59.5%:78/5%)			∖	//	ρ = −0.8878	ρ = −0.8744
							*p* = 0.0182	*p* = 0.0227
TAM ExternalControl	35.5%	(14%:57%)				∖	ρ = 0.7991	
							*p* = 0.0564	
TAM Enjoyment	78.5%	(28.5%:86%)					∖	ρ = 0.8202
								*p* = 0.0456

## Abbreviations

The following abbreviations are used in this manuscript:

API	Application Programming Interface
ARAT	Action Research Arm Test
CI	Control Interface
GUI	Graphical User Interface
EA	Electrode Array
ECS	Embedded Control System
IMU	Inertial Measurement Units
MCID	minimal clinically important difference
MI	Motricity Index
MMSE	MiniMental State Examination
NMES	NeuroMuscular Electrical Stimulation
RCT	Randomised Clinical Trial
RSSI	Received Signal Strength Indicator
SUS	System Usability Scale
TAM	Technology Acceptance Model
UID	Unique IDentifier
VE	Virtual Electrodes
